# Three-dimensional ultrastructural analysis of the interface between an implanted demineralised dentin matrix and the surrounding newly formed bone

**DOI:** 10.1038/s41598-018-21291-3

**Published:** 2018-02-12

**Authors:** Ryuichiro Tanoue, Keisuke Ohta, Yoshihiro Miyazono, Joe Iwanaga, Akihiro Koba, Toru Natori, Osamu Iwamoto, Kei-ichiro Nakamura, Jingo Kusukawa

**Affiliations:** 1 Dental and Oral Medical Centre, Kurume University School of Medicine, Kurume, Fukuoka Japan; 2 Department of Dentistry and Oral Surgery, Jyosuikai Imamura Hospital, Tosu, Saga Japan; 3 Division of Microscopic and Developmental Anatomy, Department of Anatomy, Kurume University School of Medicine, Kurume, Fukuoka Japan

## Abstract

Previous investigators have reported that transplanted demineralised dentin matrix (DDM) influences bone formation *in vivo*. However, the specific mechanism of how dentinal tubules contribute to bone formation has not been determined with regard to DDM transplantation therapy. In this study, we ultrastructurally investigated how DDM contacted the surrounding newly formed bone using a scanning electron microscopy (SEM) three-dimensional reconstruction method that is based on focused ion beam slicing and SEM (FIB/SEM). A pulverised and processed DDM derived from human teeth was implanted into rat calvarial bone defects, and a series of X-ray computed tomographic images were obtained over 12 weeks. Implants with surrounding new bone were removed and histologically examined using FIB/SEM. After obtaining objective block-face images, the target boundary face was reconstructed three-dimensionally. The osteocytes of the new bone tissue surrounding the DDM formed a network connected by their cellular processes and formed bone tissue. It is also interesting that the cellular processes of the osteocytes extended into the dentinal tubules, and that bone tissue with canaliculi had formed and filled the DDM surface.

## Introduction

Demineralised dentin matrix (DDM), a collagen material with less antigenicity to release growth factors such as bone morphogenic proteins (BMPs), is clinically applied in several domestic and overseas facilities as a bone-filling agent in the maxillofacial field^1–8^. The general production method of the DDM involves crushing and demineralising dentine, including the cementum, after removing the enamel. DDM transplantation therapy was developed after several studies by Urist and other investigators demonstrated that demineralised bone and dentine transplantation induced bone formation^9–11^.

BMPs are present in the bone matrix, osteosarcoma tissue, and dentin matrix^12,13^. In dentistry, the BMPs in dentine have been investigated, and in recent years DDM studies have focused on particle size, production, and processing methods that result in more efficient bone formation^14,15^.

Dentine contains 35% organic substances and 65% inorganic substances. Its chemical composition is very similar to that of alveolar bone (e.g., enamel, 96% inorganic substances; cementum, 50–55% organic substances and 45–50% inorganic substances)^16,17^. The major inorganic component of dentine is hydroxyapatite (HA), which is structured with low-crystalline calcium phosphate. Demineralisation is necessary because crystalline HA inhibits the release of growth factors^17^. In addition, the demineralisation process does not degenerate growth factors^17^. In the organic parts, dentine includes type I collagen and growth factors, such as BMPs. Type I collagen includes non-collagenous proteins (NCPs), such as phosphophoryn and sialoprotein, which trigger bone resorption and generation processes. Thus, the osteoconduction and osteoinduction capability of the DDM was previously demonstrated^18^. Furthermore, the DDM has antibacterial properties^19^. These findings suggest that the DDM is biocompatible and that bone forms after DDM transplantation, even between allogeneic transplants^11^.

There are two types of artificial bone substitute materials: dense and porous body materials. In dentistry, porous bodies are more commonly used because it is necessary to integrate graft material and bone tissue. Stronger fixation may be obtained by the invasion of the new bone tissue into a porous body material with an irregular surface and highly communicable pores^20^. The DDM contains many dentinal tubules, which are a unique spatial structure of dentine. The dentinal tubule is communicable; however, the size of the pore is approximately 3 μm and therefore, cell invasion is impossible. However, no previous DDM study has discussed the significance of the existence of dentinal tubules. Therefore, the relationship between dentinal tubules and new bone formation and their respective influences has not been determined.

A new method for serial sectioning and three-dimensional (3D) analytical scanning electron microscopy (SEM), called focused ion beam/SEM (FIB/SEM) tomography, has been developed^21–23^. The FIB/SEM method enables the observation of hundreds of serial sections, the reconstruction of 3D structures with high resolution, and the quantitative analysis of structural properties. We previously performed FIB/SEM tomography to investigate the structure of organelles, cells, and various tissues; and reported new findings^23–26^.

In the current study, we ultrastructurally reconstructed the interface between the dentinal tubules and new bone tissue using FIB/SEM tomography and examined the relationship between the tubules and new bone.

## Materials and Methods

All experiments were performed in accordance with the National Institutes of Health guidelines for animal research. The protocol and study were approved by the Board for Animal Experiments of the Kurume University Animal Centre (Kurume, Japan) and the Ethical Committee for Clinical Study of Kurume University (Kurume, Japan).

### Preparation of the DDM

**Figure 1:**
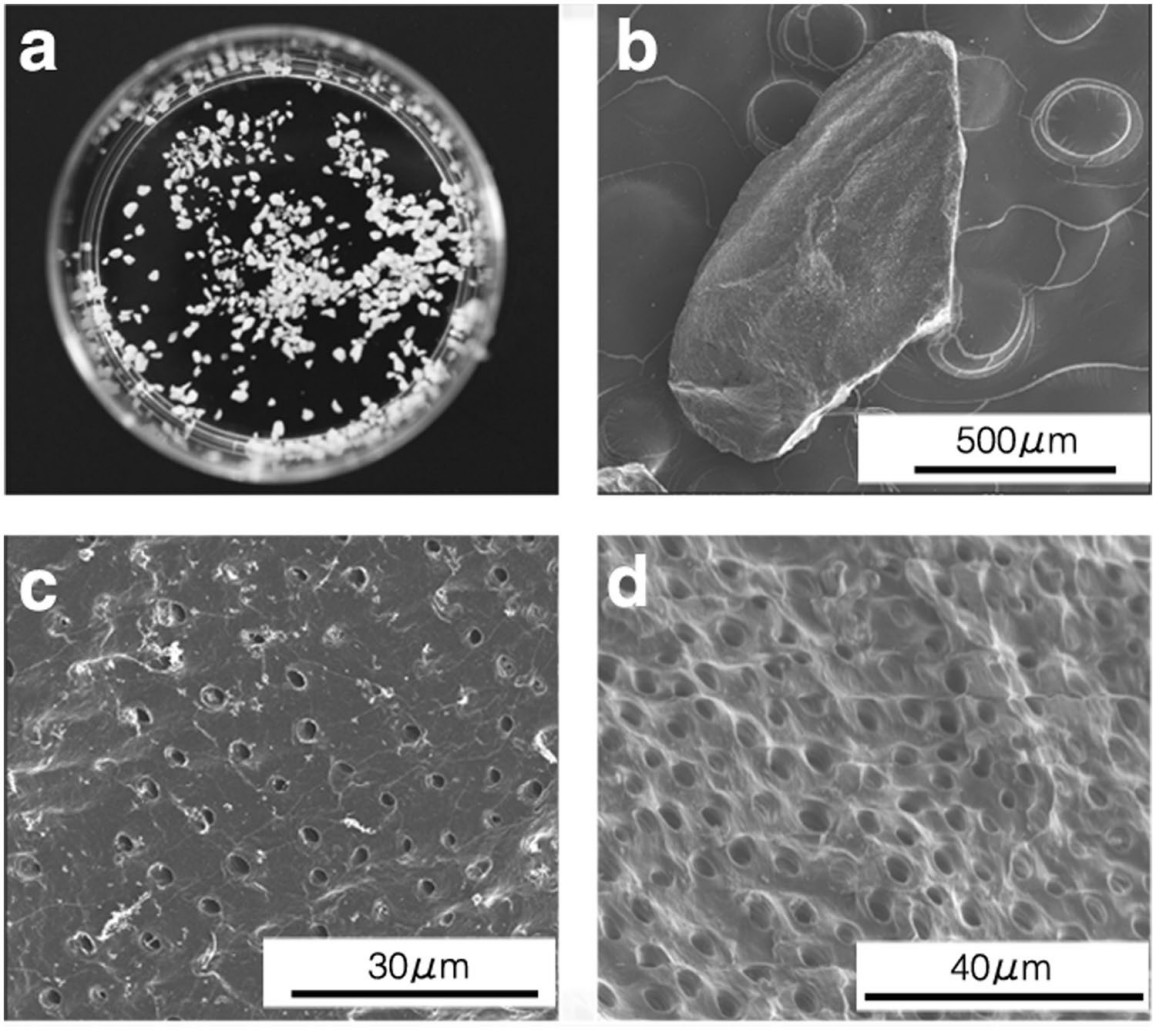
Scanning electron microscopy images of the demineralised dentin matrix (DDM). (**a**) Processed granules. (**b**) Higher magnification of image a. The surfaces of the DDM are lubricous (**c**) and rough (**d**).

Teeth extracted from healthy humans were collected from the Dental and Oral Medical Centre at Kurume University Hospital (Kurume, Japan), after obtaining informed consent from all individuals. Wisdom teeth, from which soft tissues, calculus, cavities, and tooth enamel had been removed, were crushed with saline ice using a tooth mill (Osteo-Mill; Tokyo Iken Co., Ltd, Tokyo, Japan). The particles were passed through a series of sieves (425–600 and 800–1200 μm). Approximately 500-μm particles (range, 425–600 μm) were subsequently collected and washed thoroughly in phosphate-buffered saline (PBS) containing 50 U/mL of penicillin and 50 g/mL of streptomycin (Life Technologies, Carlsbad, CA, USA). Dentin particles were completely demineralised in 2% nitric acid for 3 hours at 4 °C. They were then extensively rinsed three times in PBS for 10 minutes. After this e treatment, the DDM had one lubricous surface and one rough surface (Fig. 1).

### DDM implantation and harvest in rat calvarial bone defects

Six-week-old male Sprague–Dawley (SD) rats were purchased from Japan SLC (Hamamatsu, Japan). The rats were kept under sterile housing conditions with free access to food and water. All surgical procedures were performed after inducing intraperitoneal anaesthesia with 0.05 mg/kg pentobarbital. All efforts were made to minimise the animals’ suffering.

In this study, a rat calvarial bone defect model was used as a standard to evaluate new bone formation in osseous defects. The defects were created after inducing inhalation anaesthesia with isoflurane under high flow oxygen. First, a skin incision was formed at the centre of the parietal region, and the periosteum was continuously exfoliated. A 6-mm calvarial bone defect was formed with a trephine bur at 1000–1500 rpm under continuous saline buffer. Twenty milligrams of DDM was grafted into the defect. Another defect was created but kept empty (i.e., control). In this study, eight rats were used (n = 2 for each group). After grafting the DDM, the skin containing the periosteum was repositioned and sutured in place.

### Radiographic analysis

For some specimens, X-ray microcomputed tomography images were obtained under standardised conditions while the animals were alive at 0, 4, and 12 weeks (R_mCT2 system; Rigaku, Tokyo, Japan). Using 3D structural analysis software (TRI/3D-BON; Ratoc System Engineering, Tokyo, Japan), 3D microcomputed tomography images of the defect regions were reconstructed and used to evaluate the new bone volume.

### Statistical analysis

Statistical analysis was performed using JMP version 11 (SAS Institute Inc., Cary, NC, USA). Data are presented as the mean and standard error.

One-way analysis of variance with the Tukey–Kramer test was used to evaluate the bone regeneration at each time point, by comparing the means of the bone volume/total defect volume between the groups. Differences were significant at p < 0.05.

### Histological analysis

#### Light microscopy examination

After 12 weeks, specimens were removed from the animals under deep anaesthesia with diethyl ether and sodium pentobarbital (50 mg/kg). Briefly, six rats were transcardially perfused through the left ventricle with heparin-containing saline (10 U/mL), followed by fixing with 4% paraformaldehyde in PBS. After perfusion, calvariums containing the defect region were removed. The specimens were immersed in the same fixative for 2 h at 4 °C. They were subsequently each rinsed in buffer and decalcified in 5% ethylenediaminetetraacetate (EDTA) solution for 2 weeks at 4 °C. The EDTA solution was changed every other day. Thereafter, the specimens were trimmed, washed three times for 5 minutes in PBS, immersed overnight in PBS containing 6.8% sucrose at 4 °C, dehydrated in 100% acetone for 1 hour, and then embedded in paraffin. Five-micrometre-thick consecutive sections of the specimens were cut with a microtome. Some sections were stained with haematoxylin and eosin (HE) and toluidine blue (pH 7) for histological examination.

#### Electron microscopy examination

Another group of three rats was deeply anaesthetised with diethyl ether and sodium pentobarbital (50 mg/kg). They were transcardially perfused through the left ventricle with heparin-containing saline (10 U/mL), fixed with 2% paraformaldehyde, and then placed in 2.5% glutaraldehyde in a 0.1-M cacodylate (pH 7.3) buffer for electron microscopy. After perfusion, the calvariums containing the defect regions were removed. The specimens were immersed in the same fixative for 2 h at 4 °C. They were each rinsed in a buffer and decalcified, as described previously. After decalcification, each specimen was rinsed in the buffer three times for 10 minutes. The specimens were then cut into small cubes and subjected to a post-fixation and *en bloc* staining procedure. After three washes in the cacodylate buffer, the specimens were post-fixed for 2 hours at 4 °C in a solution containing 2% osmium tetroxide and 1.5% potassium ferrocyanide in the cacodylate buffer. The specimens were then washed three times with distilled water and immersed in 1% thiocarbohydrazide solution for 1 hour. After five washes with distilled water, they were further immersed in 2% osmium tetroxide in distilled water and washed three times with distilled water. The specimens were then stained *en bloc* in a solution of 4% uranyl acetate dissolved in a 25% methanol solution overnight for contrast enhancement and finally washed with distilled water. The specimens were then stained with Walton’s lead aspartate solution for 1 hour^27^. Afterwards, each specimen was dehydrated in an ethanol series (25%, 50%, 70%, 80%, 90%, and twice in 100%; the specimens were exposed to each percentage of ethanol for 10 minutes), followed by infiltration with an epoxy resin mixture (Epon 812; TAAB laboratories Equipment Ltd., Berks, England) and polymerisation for 72 hours at 60 °C. The surfaces of the embedded specimens were exposed using a diamond knife on the Ultracut E microtome (Leica, Wetzlar, Germany). After trimming down the resin blocks, the relevant regions were observed on a specimen holder.

Some sections of the specimens were transferred onto copper transmission electron microscopy (TEM) grids, which were conventionally stained with uranyl acetate and lead, based on Sato’s improved staining method^28^, and imaged with the H-7650 TEM system (Hitachi, Tokyo, Japan) operated at 100-kV accelerating voltage.

### FIB/SEM tomography and 3D structure reconstruction

The surface of the specimens was freshly exposed using an ultramicrotome and examined using FIB/SEM tomography (Quanta 3D FEG; FEI, Hillsboro, OR, USA). Serial images of the block face were acquired by repeated cycles of sample surface milling and imaging using the Slice & View G2 operating software (FEI). The milling was accomplished using a gallium ion beam at 30 kV with a current of 15 nA. The milling pitch was set to 100 nm/step and 420 cycles. The images were acquired at a landing energy of 2.5 keV. Other acquisition parameters were as follows: beam current, 51 pA; dwell time, 6 μs/pixel; image size, 2,048 × 1,768 pixels; 36 nm/pixel. Under the conditions of our study, the slice and view process was reapeated approximately 11 times. The resultant image stack, segmentation, and 3D reconstruction were processed using open software from Fiji (available at http://fiji.sc/Fiji)^29^ and Avizo 8.1 software (FEI Visualization Science Group, Burlington, MA, USA). Images could be observed with optional x-y-z plane sectioning. After reconstruction, the interface between the transplanted DDM and surrounding new bone was measured. The interface between the DDM and the dentinal tubules, and the osteocyte network and morphology were recorded and calculated using the Avizo software (FEI Visualization Science Group). Furthermore, the DDM surface (Fig. 1) was recorded using the SEM function.

### Energy-dispersive X-ray spectrometry analysis

The surfaces of non-decalcified specimens (n = 6) of implanted DDMs were polished and observed on a block-face image (BFI) using SEM. Energy-dispersive X-ray spectrometry was used to estimate the mineral components on the outside and inside of the dentinal tubule, using the Quantax 70 system (Bruker Nano GmbH, Berlin, Germany). The DDM specimens before implantation were used as the control.

## Results

### Microcomputed tomography assessment

**Figure 2:**
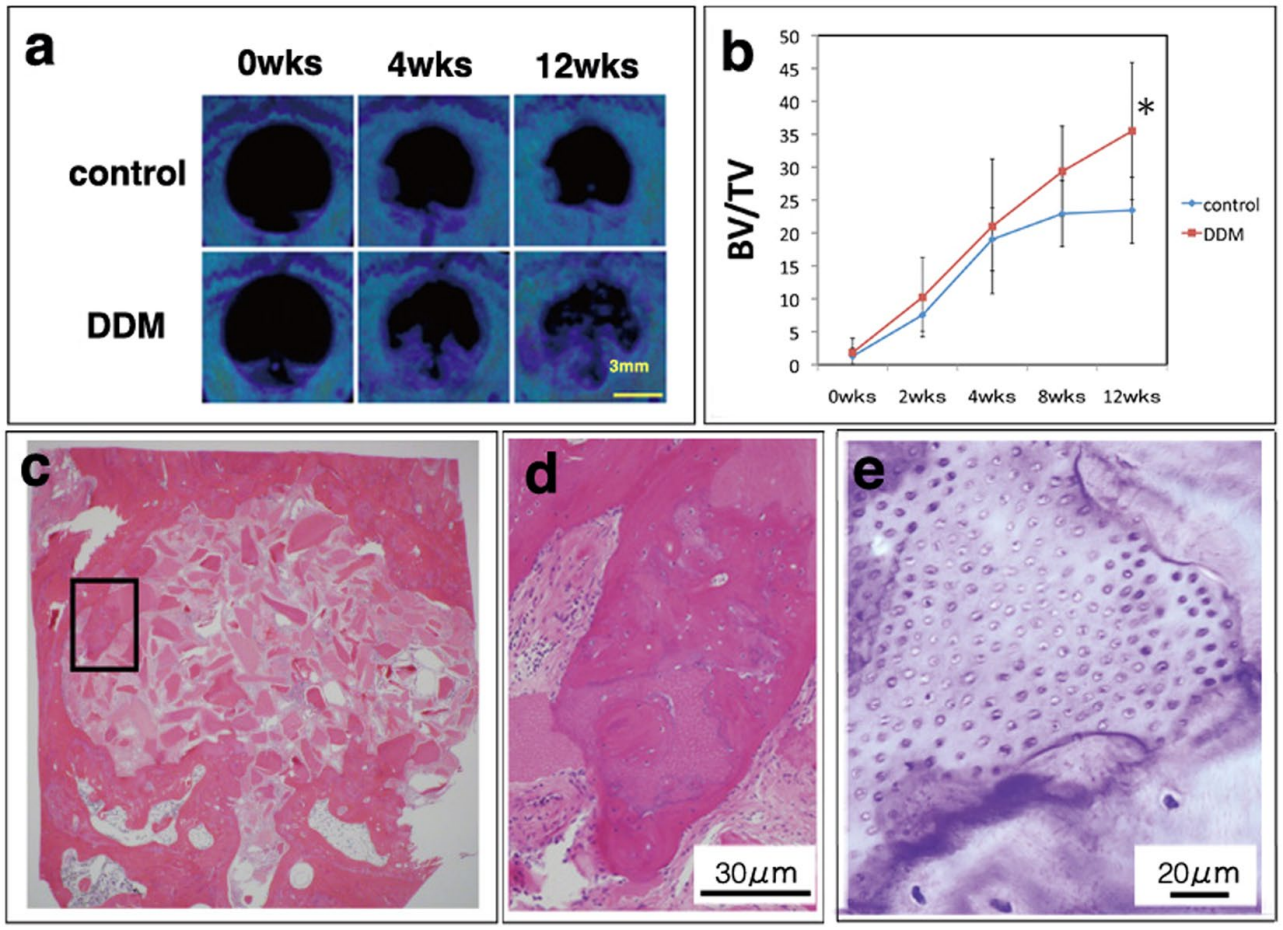
Computed tomography assessment and light micrographs. (**a**) At 0 weeks, no radiopacity was visible in the defect regions in the demineralised dentin matrix (DDM)-implanted and control groups. Over 12 weeks, the defect region showed increasing radiopacity in the DDM-implanted group. (**b**) The bone volume/total defect volume (BV/TV) increased incrementally. At 12 weeks, there was a significant difference. (**c**) The horizontal plane of the defect area in the DDM group at 12 weeks. (**d**) Higher magnification of the square area in panel (c). Newly formed bone tissue with the DDM as the core is visible in the haematoxylin and eosin stained sections from the DDM-implanted group. (**e**) A serial section of the image in (**d**). Solid red–purple matrices of the dentinal tubules are visible in the toluidine blue-stained sections.

Immediately after surgery, no radiopacity existed in the defect regions in the DDM-implanted group because a completely DDM was implanted. Radiopacity of the defect region increased in the DDM-implanted group over 12 weeks, and the bone volume/total defect volume also increased incrementally (Fig. 2a). However, this parameter in the control group showed no significant increase. The control group had only a small increase in radiopacity in the margin of the native bone. At 12 weeks, each group showed significant differences in the amount filled with newly formed bone tissue (Fig. 2b).

### Light microscopy assessment

A small amount of lone DDM remained in the centre of the defect region. However, substantial newly formed bone tissue with the DDM as the core was noted. Eosinophilic material, which contained osteocytes, was observed in HE-stained sections from the DDM-implanted group (Figs 2c and d). In addition, toluidine blue-stained sections contained solid red–purple matrices of the dentinal tubules (Fig. 2e).

### Three-dimensional ultrastructural analysis using FIB/SEM tomography

**Figure 3:**
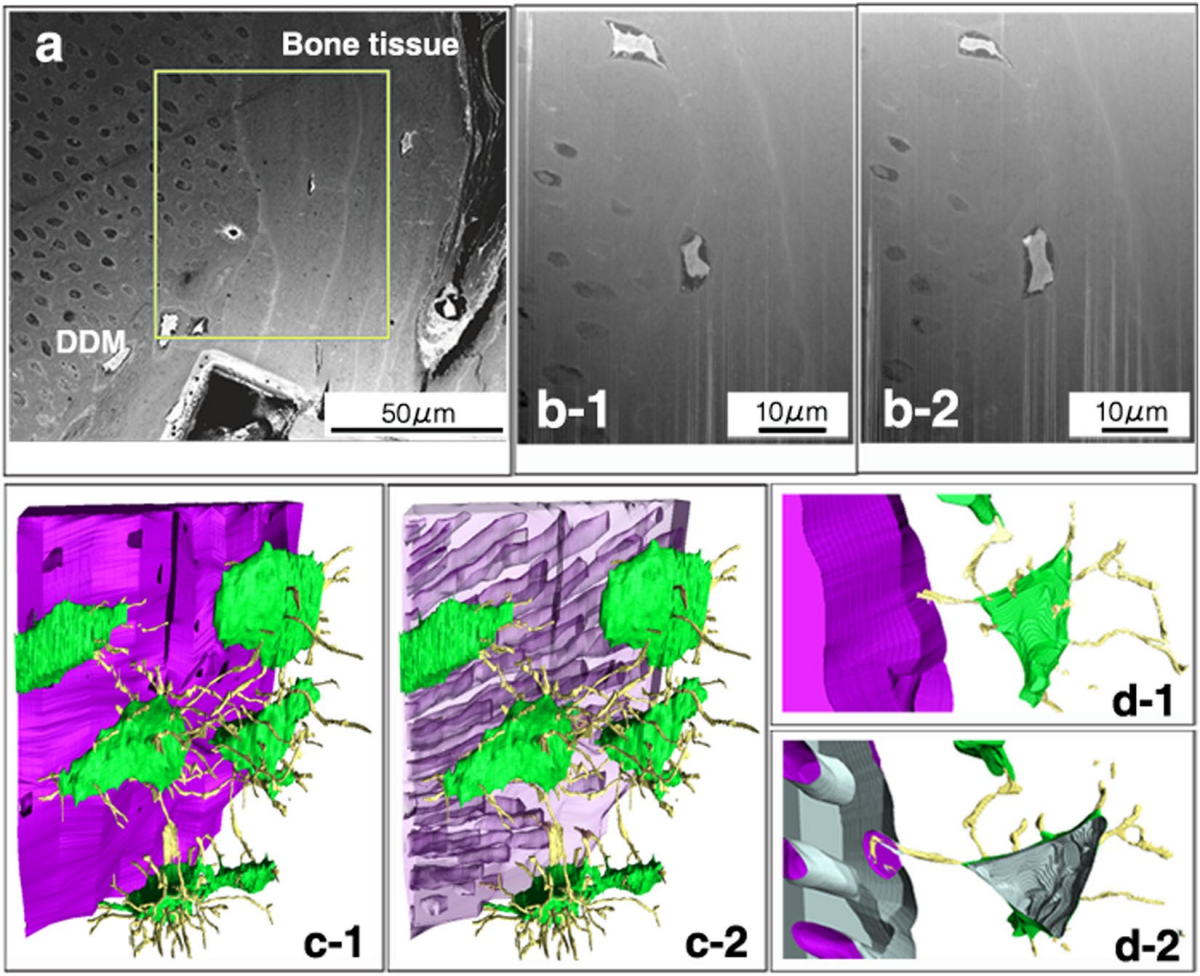
Focused ion beam/scanning electron microscopy (FIB/SEM) tomography and three-dimensional (3D)-structure reconstruction. (**a**) The FIB/SEM tomography image of the interface between the implanted demineralised dentin matrix (DDM) and surrounding new bone. The left side shows the DDM, and the right side shows the newly formed bone tissue. (**b**) Higher magnification of the square area in panel (**a**). Some osteocytes surround the DDM. Images b-1 and b-2 are serial sections. (**c**) The 3D reconstruction image of the square area. Image c-2 is the DDM skeleton image of image c-1. The 3D reconstruction image confirms that osteocytes form a network with cell processes. (**d**) Higher magnification of the interface between the DDM and new bone in the 3D reconstruction image. Image d-2 is the torn section of image d-1. Some cell processes of the osteocytes extend into the dentinal tubules in the torn surface.

FIB/SEM tomography allowed the 3D reconstruction of the interface between the implanted DDM and surrounding new bone. The target milling area, which is enclosed by a square in Fig. 3a, was acquired with a gallium ion beam. In Fig. 3a, the left side shows the DDM, and the right side shows the newly formed bone tissue. Some osteocytes surrounded the DDM, as shown in Fig. 2b. This is a limitation of 2D images. However, the 3D reconstruction images could confirm that the cell processes of the osteocytes formed a network (Fig. 3c). Furthermore, some cell processes of these osteocytes extended into the dentinal tubules (Fig. 3d). We also confirmed that the bone tissue with microscopic bone tubules invaginated the dentinal tubules on the DDM surface.

### Morphological assessment of dentinal tubules by electron microscopy

**Figure 4:**
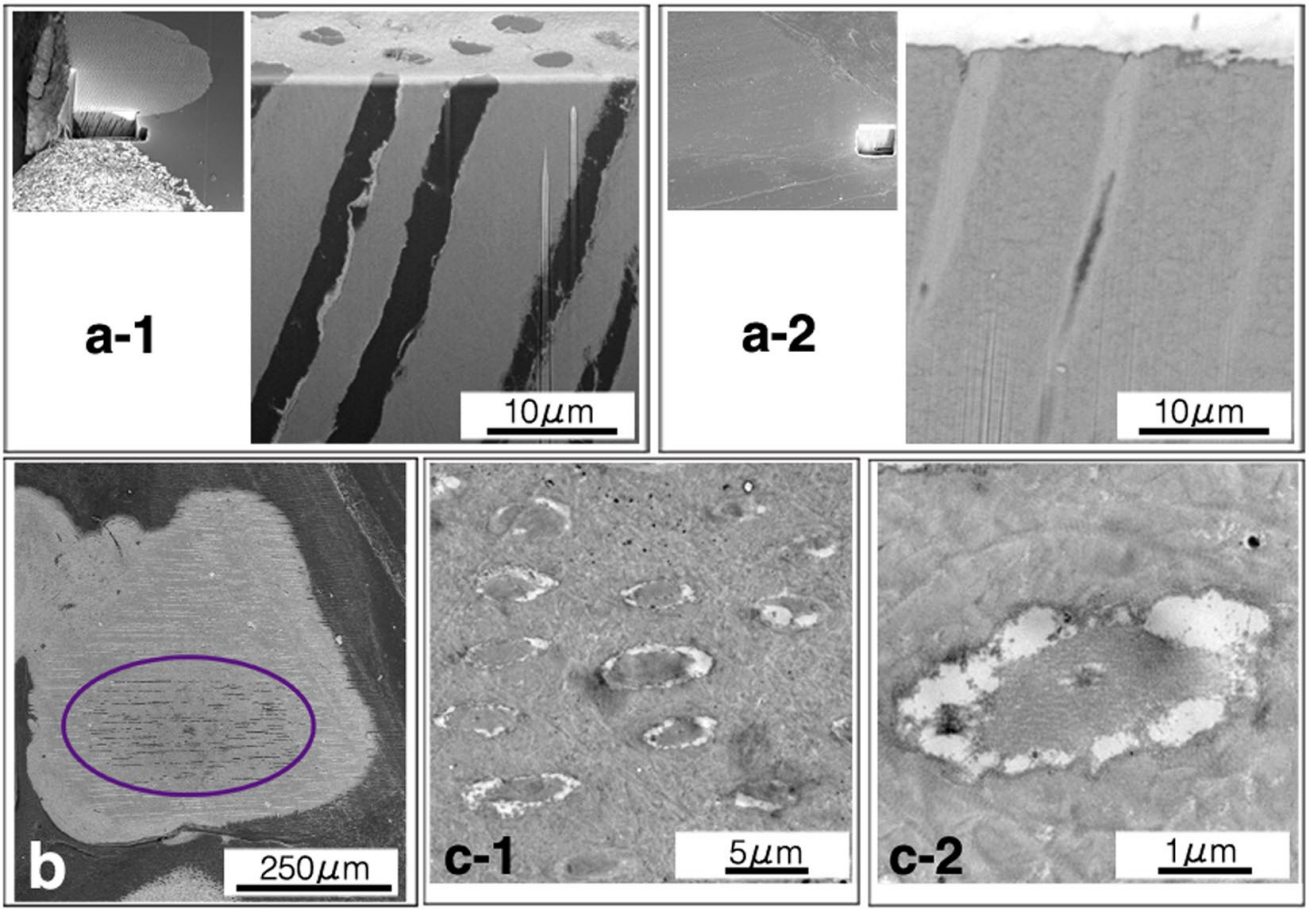
Observation of block-face images (BFIs) and transmission electron microscopy (TEM) images. (**a**) BFIs of the dentinal tubules. The left images in a-1 and a-2 are at low magnification. Image a-1 shows low electron density regions in the dentinal tubules of non-implanted demineralised dentin matrix (DDM) particles. Image a-2 shows high electron density regions in the dentinal tubules of the implanted DDM particles. (**b**) A BFI of a DDM granule. The BFI image of the implanted DDM is at low magnification. In this image, the electron density of the dentinal tubules in the centre of the DDM (encircled area) is lower than that of the tubules on the outside of the DDM particles. The newly formed bone tissue surrounds the DDM. (**c**) Transmission electron microscopy images of the horizontal plane of the dentinal tubules. (c-1) The high electron density regions are observed within the dentinal tubules. Bone-like tissue is visible. (c-2) Higher magnification of image c-1. The processes tissue, which contrasts with the bone-like tissue, is in the centre of bone-like tissue.

In the BFI, low electron density regions were contained in the dentinal tubules of non-implanted DDM particles, whereas high electron density regions were contained in the dentinal tubules of the implanted DDM particles (Fig. 4a). On a low-magnification BFI image, the electron density of the dentinal tubules in the centre of the DDM was lower than that of the tubules outside the DDM particles (Fig. 4b). Bone-like tissue was observed on the TEM horizontal plane study of the high electron density regions within the dentinal tubules (Fig. 4c). The process tissue, which contrasts with the bone-like tissue, was in the centre of the bone-like tissue.

### Elemental analysis of dentinal tubules using energy-dispersive X-ray spectrometry

**Figure 5:**
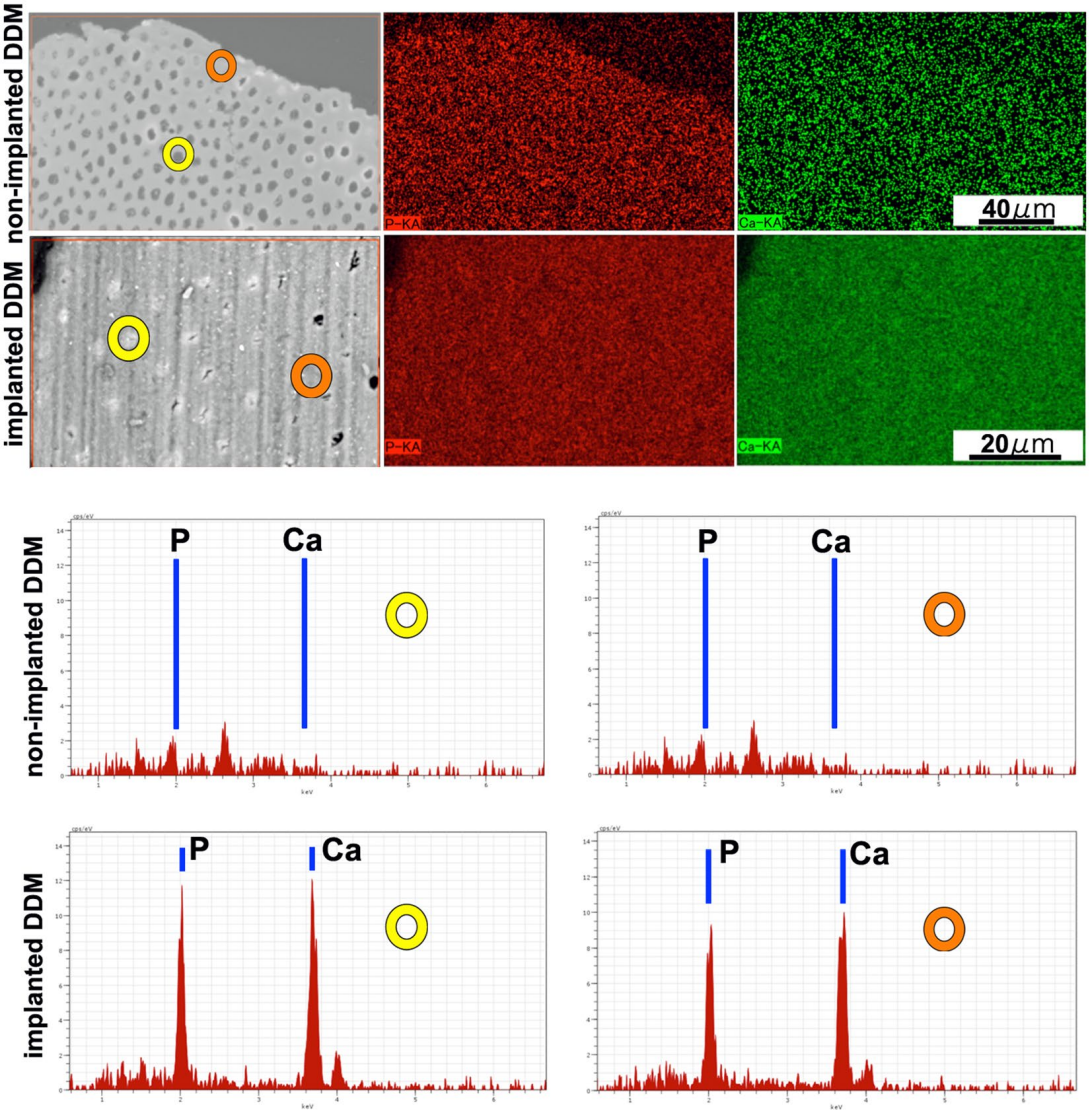
Elemental analysis of dentinal tubules, based on energy-dispersive X-ray spectrometry. The yellow circles indicate the dentinal tubules, and the orange circles indicate the matrix. The Ca and P levels are higher in the implanted demineralised dentin matrix (DDM) particles than in the non-implanted DDM particles on the polished surface. The Ca and P levels are higher in the dentinal tubules than in the matrix.

The Ca and P levels were higher in the implanted DDM particles than in the non-implanted DDM particles on a polished surface (Fig. 5). In addition, the Ca and P levels were higher in the dentinal tubules than in the matrix. However, the Ca and P levels were low in all parts of the DDM before implantation.

## Discussion

In this study, we successfully reconstructed the ultrastructural interface between the dentinal tubules and new bone tissue using FIB/SEM tomography. Through this technique, we found that the osteocytes of the new bone tissue surrounding the DDM formed a network connected by the cellular processes and formed bone tissue and that the cellular processes of the osteocytes extended into the dentinal tubules. The DDM used in this study was completely demineralised; therefore, it is possible that bone growth factors, such as BMPs, were contained only in small amounts in this completely DDM. Koga reported that partial DDM transplantation induced more bone formation than did complete DDM transplantation^14^. Hence, the DDM used in this study may have induced osteoconduction, depending on the presence of collagen, and the osteoinductive potential may have been low. As a result, the amount of bone formation in the DDM-implanted group was not prominent, and it took 12 weeks to achieve a significant difference, compared with the control group. A partial DDM (particle size: 1000 μm) is the most efficient as a preparatory condition for bone formation^14^. In a partial DDM, because more BMPs remain in the matrix, more bone may be formed than was observed in this study. However, we used a complete DDM in our study because we wanted to estimate the ossification of the DDM itself. A DDM with a 500-μm particle size was prepared for FIB/SEM and other electron microscopic techniques. In this defect model, substantially more new bone tissue may have formed if the demineralisation time was shorter and a DDM with a larger particle size was transplanted.

Dentine, including the DDM, contains many dentinal tubules, which is a unique spatial structure. The dentinal tubules run radially from the pulp to the enamel dentin border and are involved in the mechanism of tooth stimulation. The odontoblast processes and interstitial fluids are included in the tubules to form and maintain the dentine. Human dentine contains 20,000–60,000 dentinal tubules per cubic millimetre^30^. The diameter of the tubules in dentine near to the pulp is larger than that in other layers; its maximum diameter is approximately 3 μm. The dentinal tubules in the BFI may have been near the pulp cavity because their diameter was approximately 3 μm. Li *et al*. reported that the demineralised dentinal tubules would be wider than normal^31^. In the current study, the TEM and BFI images showed that the structural form of the dentinal tubules remained consistent after demineralisation, compared with that of non-demineralised dentinal tubules. The structure of DDM dentinal tubules after transplantation, even when they were calcified, was also preserved.

The significance of the porosity of an artificial bone material was described in a previous study and is extremely important for tissue fitting^20^. The pore diameters must be sufficiently large to allow cell entry. To increase contact with the surrounding tissue, in some cases, dentine is processed into a shape other than granules, and the pores are formed by a dental bur to facilitate the invasion of cells and blood vessels^2,17^. Kuboki *et al*. reported that the geometric structure of the implant is important for bone–cartilage induction^32^. Mutrata *et al*. reported that bone is preferentially induced when a porous apatite with a pore size of 150 μm, which does not prevent the invasion of cells and blood vessels, is used as a carrier for BMPs^33^. The size of mesenchymal cells, such as osteoblasts and bone cells, ranges from 10 to 20 μm, and the size of osteoclasts ranges from 20 to 100 μm or larger. Therefore, it is natural to assume that cell penetration into dentinal tubules is impossible. In this study, we did not confirm the invasion of whole osteocytes into the dentinal tubules on the DDM surface. However, it is interesting that the invasion of osteocyte processes and cytoplasm occurred. Under these conditions, the degree of invasion was approximately 5 μm from the DDM–new bone interface. We also confirmed that the new bone tissue, with microscopic bone tubules, invaginated the dentinal tubules on the DDM surface and combined with each other. The bonding form was an uneven and concave–convex boundary structure. The finding suggests that the existence of dentinal tubules is an important advantage of using the DDM. Our energy-dispersive X-ray spectroscopy approach revealed higher levels of Ca and P in the dentinal tubules than in the matrix. The high-density tissue formed in the dentinal tubules was different from the simple surrounding calcified tissue and was continuous with the surrounding bone tissue. This finding sufficiently illustrates bone tissue formation in dentinal tubules.

**Figure 6:**
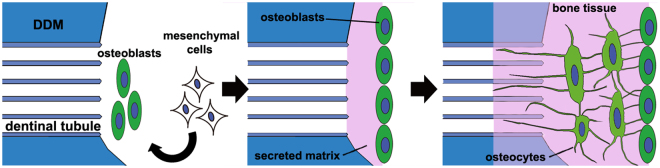
A schematic of the new-bone formation process after demineralised dentin matrix (DDM) transplantation.

We believe that bone formation after DDM transplantation occurs as follows (Fig. 6). When the DDM is transplanted into the bone defect area, a small amount of BMPs that are gradually released from the DDM, induce mesenchymal cells to differentiate into osteoblasts. The osteoblasts secrete the matrix, which is mineralised, and forms a new bone. In the process, the osteoblasts differentiate into osteocytes, which are buried in mineralised tissues. The processes of osteocytes then form a network on the DDM surface, some of them extending into the dentinal tubules. The matrix is mineralised by causing the mineralisation of type I collagen through the decomposition of pyrophosphate, due to alkaline phosphatase expression by osteoblasts^34^. Ninety percent of the organic substances in the DDM is type I collagen. Therefore, it is possible that the higher mineralisation around the pores of dentin tubules is caused by the DDM itself being mineralised because of the influence of the surrounding osteoblasts and bone formation. Additionally, the intertubular dentine is mineralised by phosphophoryn, which is the most abundant NCP in dentine^18^. Finally, a new bone is formed and added around the DDM, and it is predicted that the DDM is later replaced by bone.

The DDM is biocompatible and has excellent capabilities, such as osteoconduction and osteoinduction. Smith *et al*. reported that dentine showed antibacterial activity against three types of anaerobic bacteria associated with dental disease^19^. Homogenous and allogeneic DDM experiments report the induction of bone formation without host immune rejection^9^. In the present study, DDM transplantation after acid treatment revealed no specific presence of phagocytic cells around the DDM and new bone, despite the transplantation of human teeth into rats. The DDM was also not rejected from recipients. Kim *et al*. reported that osteoclasts absorb the DDM margin and show remodelling^3^. In this study, we found no evidence of osteoclast remodelling. In the future, we plan to increase the number of samples for further observation; change the experimental timing; and pursue phagocytic cells, including osteoclasts, using FIB/SEM.

In the present study, we demonstrated that a new bone around the implanted DDM formed directly on the DDM surface and inside the dentinal tubules via their processes, and its morphology was a concave–convex boundary bonding structure. We believe that this process is very important to bone formation from a therapeutic and functional perspective. We hope that these findings and the FIB/SEM tomography method will help bone regeneration therapy.

### Data availability

The data that support the present findings are available from *Scientific Reports*. However, restrictions apply to the availability of these data, which were used under license for the current study, and therefore are not publicly available. Data are, however, available from the authors on reasonable request and with the permission of *Scientific Reports*.

## References

[1] Murata, M. *et al*. Autograft of dentin materials for bone regeneration. *Advances in Biomaterials Science and Biomedical Applications* (ed. Rosario, P.), https://www.intechopen.com/books/advances-in-biomaterials-science-and-biomedical-applications/autograft-of-dentin-materials-for-bone-regeneration (2013).

[2] Kabir MA, Murata M, Kusano K, Akazawa T, Shibata T (2015). Autogenous demineralized dentin graft for third molar socket regeneration a case report. Dentistry..

[3] Kim YK (2010). Development of a novel bone grafting material using autogenous teeth. Oral Surg Oral Med Oral Pathol Oral Radiol Endod..

[4] Lee EY, Kim ES, Kim KW (2014). Scanning electron microscopy and energy dispersive X-ray spectroscopy studies on processed tooth graft material by vacuum-ultrasonic acceleration. Maxillofac Plast Reconstr Surg..

[5] Lee JY, Kim YK, Yi YJ, Choi JH (2013). Clinical evaluation of ridge augmentation using autogenous tooth bone graft material: case series study. J Korean Assoc Oral Maxillofac Surg..

[6] Gomes MF, Abreu PP, Morosolli AR, Araujo MM, Goulart MD (2006). Densitometric analysis of the autogenous demineralized dentin matrix on the dental socket wound healing process in humans. Braz Oral Res..

[7] Kim SY, Kim YK, Park JC, Ku JK, Yun PY (2017). Evaluation of efficacy of demineralised dentin matrix fixed with recombinant human bone morphogenetic protein-2. J Oral Maxillofac Surg..

[8] Murata M (2017). Histological evidences of dentin autograft for bone regeneration. J Oral Maxillofac Surg..

[9] Urist MR (1965). Bone: formation by autoinduction. Science..

[10] Yemans JD, Urist MR (1967). Bone induction by decalcified dentine implanted into oral, osseous and muscle tissues. Arch Oral Biol..

[11] Bang G, Urist MR (1967). Bone induction in excavation chambers in matrix of decalcified dentin. Arch Surg..

[12] Hanamura H (1980). Solubilized bone morphogenetic protein (BMP) from mouse osteosarcoma and rat demineralized bone matrix. Clin Orthop Relat Res..

[13] Butler WT, Mikulski A, Urist MR, Bridges G, Uyeno S (1977). Noncollagenous proteins of a rat dentin matrix possessing bone morphogenetic activity. J Dent Res..

[14] Koga T (2016). Bone regeneration using dentin matrix depends on the degree of demineralization and particle size. PLoS One..

[15] Kabir MA (2017). Evaluation of perforated demineralized dentin scaffold on bone regeneration in critical-size sheep iliac defects. Clin Oral Impl Res..

[16] Kim YK (2011). Analysis of the inorganic component of autogenous tooth bone graft material. J Nanosci Nanotechnol..

[17] Kim YK (2013). Tooth-derived bone graft material. J Korean Assoc Oral Maxillofac Surg..

[18] Ritchie HH, Ritchie DG, Wang LH (1998). Six decades of dentinogenesis research. Historical and prospective views on phosphophoryn and dentin sialoprotein. Eur J Oral Sci..

[19] Smith JG, Smith AJ, Shelton RM, Cooper PR (2012). Antibacterial activity of dentine and pulp extracellular matrix extracts. Int Endod J..

[20] Habibovic P, Sees TM, van den Doel MA, van Blitterswijk CA, de Groot K (2006). Osteoinduction by biomaterials-physiochemical and structural influences. J Biomed Mater Res A.

[21] Heymann JA (2006). Site-specific 3D imaging of cells and tissues with a dual beam microscope. J. Struct. Biol..

[22] Knott G, Marchman H, Wall D, Lich B (2008). Serial section scanning electron microscopy of adult brain tissue using focused ion beam milling. J. Neurosci..

[23] Ohta K (2012). Beam deceleration for block-face scanning electron microscopy of embedded biological tissue. Micron..

[24] Kanazawa T (2016). Histomorphometric and ultrastructural analysis of the tendon-bone interface after rotator cuff repair in a rat model. Sci. Rep..

[25] Hirashima S (2015). Anchoring structure of the calvarial periosteum revealed by focused ion beam/scanning electron microscope tomography. Sci. Rep..

[26] Yoshitomi M (2016). Three-dimensional ultrastructural analyses of anterior pituitary gland expose spatial relationships between endocrine cell secretory granule localization and capillary distribution. Sci. Rep..

[27] Walton J (1979). Lead aspartate, an en bloc contrast stain particularly useful for ultrastructural enzymology. J Histochem Cytochem..

[28] Takagi I (1990). Penetration and stainability of modified Sato’s lead staining solution. J Electron Microsc..

[29] Schindelin J (2012). Fiji: an open-source platform for biological-image analysis. Nat Methods..

[30] Schilke R, Lisson JA, Bauss O, Geurtsen (2000). Comparison of the number and diameter of dentinal tubules in human and bovine dentine by scanning electron microscopic investigation. Arch Oral Biol..

[31] Li R (2011). Human treated dentin matrix as a natural scaffold for complete human dentin tissue regeneration. Biomaterials..

[32] Kuboki Y (1995). Two distinctive BMP-2 carriers induce zonal chondrogenesis and membranous ossification, respectively; geometrical factors of matrices for cell-differentiation. Connect Tissue Res..

[33] Murata M, Inoue M, Arisue M, Kuboki Y, Nagai N (1998). Carrier-dependency of cellular differentiation induced by bone morphogenetic protein in ectopic sites. Int J Oral Maxillofac Surg..

[34] Murshed M, Harmey D, Millan JL, McKee MD, Karsenty G (2005). Unique coexpression in osteoblasts of broadly expressed genes accounts for the spatial restriction of ECM mineralization to bone. Genes Dev..

